# Structural and optical characterization of pure Si-rich nitride thin films

**DOI:** 10.1186/1556-276X-8-31

**Published:** 2013-01-16

**Authors:** Olivier Debieu, Ramesh Pratibha Nalini, Julien Cardin, Xavier Portier, Jacques Perrière, Fabrice Gourbilleau

**Affiliations:** 1 CIMAP, UMR 6252 CNRS-ENSICAEN-CEA-UCBN, Ensicaen, 6 Bd Maréchal Juin, 14050 Caen, cedex 4, France; 2 UNIV PARIS 06, INSP NANOSCIENCE PARIS, CNRS, UMR 7588, 75015 Paris, France

**Keywords:** Silicon nitride, Silicon nanocrystals, Amorphous silicon nanoparticles, FTIR, Raman, XRD, Laser annealing, Photoluminescence

## Abstract

The specific dependence of the Si content on the structural and optical properties of O- and H-free Si-rich nitride (SiN_*x*>1.33_) thin films deposited by magnetron sputtering is investigated. A semiempirical relation between the composition and the refractive index was found. In the absence of Si-H, N-H, and Si-O vibration modes in the FTIR spectra, the transverse and longitudinal optical (TO-LO) Si-N stretching pair modes could be unambiguously identified using the Berreman effect. With increasing Si content, the LO and the TO bands shifted to lower wavenumbers, and the LO band intensity dropped suggesting that the films became more disordered. Besides, the LO and the TO bands shifted to higher wavenumbers with increasing annealing temperature which may result from the phase separation between Si nanoparticles (Si-np) and the host medium. Indeed, XRD and Raman measurements showed that crystalline Si-np formed upon 1100°C annealing but only for SiN_*x*<0.8_. Besides, quantum confinement effects on the Raman peaks of crystalline Si-np, which were observed by HRTEM, were evidenced for Si-np average sizes between 3 and 6 nm. *A contrario*, visible photoluminescence (PL) was only observed for SiN_*x*>0.9_, demonstrating that this PL is not originating from confined states in crystalline Si-np. As an additional proof, the PL was quenched while crystalline Si-np could be formed by laser annealing. Besides, the PL cannot be explained neither by defect states in the bandgap nor by tail to tail recombination. The PL properties of SiN_*x*>0.9_ could be then due to a size effect of Si-np but having an amorphous phase.

## Background

Since the discovery of efficient visible photoluminescence (PL) of silicon nanoparticles (Si-np) due to quantum confinement effects (QCE) [1], the possibility of bandgap engineering of Si-based materials through the Si-np size control makes Si-based nanostructured material attracting for future applications in optoelectronics as low-cost, miniaturized, and CMOS-compatible, light-emitting devices (LEDs), laser, as well as photovoltaic devices. In the past, researches were focused on luminescent Si-np embedded in Si oxide media. However, the insulating nature of Si oxide remains a barrier for the production of future electrically pumped LEDs and efficient photovoltaic cells. This detrimental aspect can be overcomed to an extent, using a higher conductive host medium like Si nitride which has a lower bandgap energy than SiO_2_.

The first results on Si nitride are promising since many researchers have reported on efficient visible PL with tunable light emission via the change of the Si nitride composition. However, it also turns out that N-rich nitride [2-4] and Si-rich nitride thin films containing amorphous [5-8] or crystalline [9-14] Si-np or without Si-np [15-18] can exhibit PL in the same spectral range. As a result, the mechanism of the PL in Si nitride is still a controversial subject in the literature. QCE in amorphous or crystalline Si-np, defect states in the bandgap, and band tail recombination have been proposed to account for the PL. However, since the synthesis methods were mostly based on chemical vapor deposition techniques, most of the films contained a significant amount of hydrogen [2,5,8,10,11,13,14,16] and, in some cases, of oxygen [19,20], which can both contribute to the PL. Consequently, it is difficult to experimentally distinguish the mechanisms of the PL.

Then, this article is significant since we report on the structural and optical properties of Si-rich SiN_*x*<1.33_ thin films devoid of hydrogen and oxygen. The films were deposited by radio frequency (RF) magnetron sputtering. The excess of Si incorporated during the sputtering process makes possible the formation of Si-np during a suitable annealing. The microstructural properties of the films with regard to the composition and the annealing temperature are investigated. The possible contributions of the Si nitride medium and of Si-np formed during thermal annealing, or laser annealing, on the origin of the PL are discussed notably as a function of the Si-np phase (crystalline or amorphous).

## Methods

In this work, pure amorphous Si-rich SiN_*x*_ thin films were deposited on p-type 250-μm-thick (100) Si wafers and on fused silica substrates by two methods of RF magnetron sputtering using argon as the main sputtering gas. The films were deposited either by N_2_-reactive sputtering of a Si target or by co-sputtering of Si_3_N_4_ and Si targets. The Si content was monitored either by the N_2_/Ar partial pressure ratio (≡Ar/N_2_) or by the RF target power ratio P_Si_/(P_Si_ + P_Si3N4_) ≡ Si/Si_3_N_4_. The grown temperatures were 200°C and 500°C, and the plasma pressures were 2 and 3 mTorr. We adjusted the deposition time to ensure that the films thicknesses were of the same order of magnitude (100 to 200 nm) in order to avoid any effect on the optical and structural properties. The films were subsequently annealed in a N_2_ gas flow in a tubular furnace during 1 h.

The layer compositions were determined by Rutherford backscattering spectrometry (RBS). RBS measurements were carried out at room temperature using a 1.9 MeV ^4^He^+^ ion beam with an incident direction normal to the sample surface. The backscattered ions were collected at a scattering angle of 165°. The analysis of the RBS spectra, which were performed using the simulation code SIMNRA [21], enables us to quantify (a) the atomic fraction of the various elements with an accuracy of 0.8 at.% for Si and N and 0.2 at.% for Ar and (b) to determine the atomic areal densities of the films. The infrared absorption properties were investigated by means of a Thermo Nicolet (Nexus model 670) Fourier transform infrared (FTIR) spectrometer. The band positions were obtained by fitting the data with Gaussians. The film microstructure was investigated by Raman spectroscopy with a 532-nm continuous-wave laser illumination with a spot diameter of 0.8 μm. Several neutral density filters were employed to tune the excitation power density from 0.14 to 1.4 MW/cm^2^. A dispersive Horiba Jobin-Yvon Raman spectrometer with a resolution of 1.57 cm^−1^, equipped with a confocal microprobe and a CCD camera, was used to acquire the Stokes scattering spectra of the thin layers that were exclusively deposited on fused silica substrates. We also studied the film microstructure by X-ray diffraction (XRD) using a Phillips X’PERT HPD Pro device with Cu K_*λ*_ radiation (*λ* = 0.1514 nm) at a fixed grazing incidence angle of 0.5°. Asymmetric grazing geometry was chosen to increase the material volume interacting with the X-ray beam and to eliminate the contribution of the Si substrate. Moreover, the structure was investigated by high-resolution transmission electron microscopy (HRTEM) on cross-sectional samples using a JEOL 2010F (200 kV) microscope.

The optical properties of the films were investigated by spectroscopic ellipsometry using a Jobin-Yvon ellipsometer (UVISEL) with an incident angle of 66.2°. The experimental data were fitted by a dispersion law based on the Forouhi-Bloomer model for amorphous semiconducting and insulating materials [22] using the DeltaPsi2 software [23] which determines the refractive index *n*, the absorption coefficient *α* versus the photon energy, and the layer thicknesses. The PL spectra were measured using the 457-nm lines of an Ar^+^ ion laser (12.7 W/cm^2^) and a fast Hamamatsu photomultiplier after dispersion of the light in a Jobin-Yvon TRIAX-180 monochromator. The PL measurements were corrected from the spectral response of the PL setup.

## Results

We first report on the combined analysis of the SiN_*x*_ film composition by RBS and ellipsometry. Then, the microstructure and the optical properties of the films are investigated as a function of the composition, as well as the annealing temperature.

### RBS

**Figure 1:**
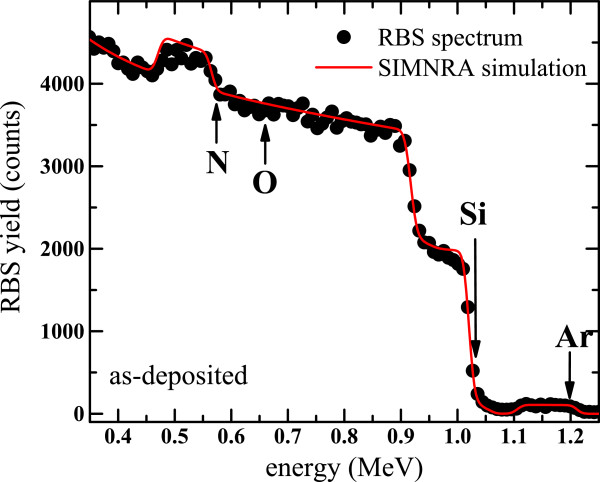
**RBS spectrum of a SiN**_***x***_**layer with the corresponding SIMNRA simulation curve.** The film was deposited on a Si substrate by the N_2_-reactive method. Surface peaks of N, O, Si, and Ar are indicated by arrows.

Figure 1 shows a typical RBS spectrum of a SiN_*x*_ layer with the corresponding simulation curve obtained using the SIMNRA code with a composition of 49.8, 48.6, and 1.6 at.% of Si, N, and Ar, respectively. The presence of residual Ar attests that the film is as-deposited. Interestingly, no oxygen was detected in all RBS spectra whatever the synthesis method, suggesting that the films do not contain oxygen or less than the detection threshold (0.2 at.%).

### Ellipsometry

**Figure 2:**
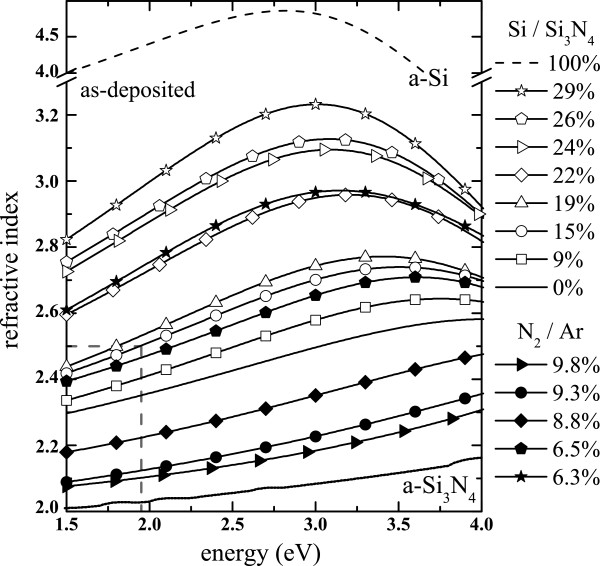
**Evolution of the dispersion curves of SiN**_***x***_**thin films.** The films were produced by the N_2_-reactive (full symbols) and the co-sputtering (empty symbols) methods as a function of the Ar/N_2_ gas flow ratio and the Si/Si_3_N_4_ target power ratio, respectively. The dispersion curve of Si_3_N_4_ from [28] is shown for comparison.

Figure 2 shows the evolution of the dispersion curves of SiN_*x*_ films deposited on Si wafer by the co-sputtering and N_2_-reactive methods with the synthesis parameters Si/Si_3_N_4_ and N_2_/Ar, respectively. The dispersion curves progressively change from the one of stoichiometric amorphous Si nitride (a-Si_3_N_4_) to that of amorphous Si (a-Si) with increasing Si/Si_3_N_4_ or decreasing N_2_/Ar. This evolution is due to the only increase of the Si incorporation during the growth, which is explained by the drop of the amount of reaction between N_2_ and Si for the N_2_-reactive method, and by the increase of the Si content into the plasma for the co-sputtering method. Indeed, one can notice that the dispersion curves change in the same way independently of the deposition method.

**Figure 3:**
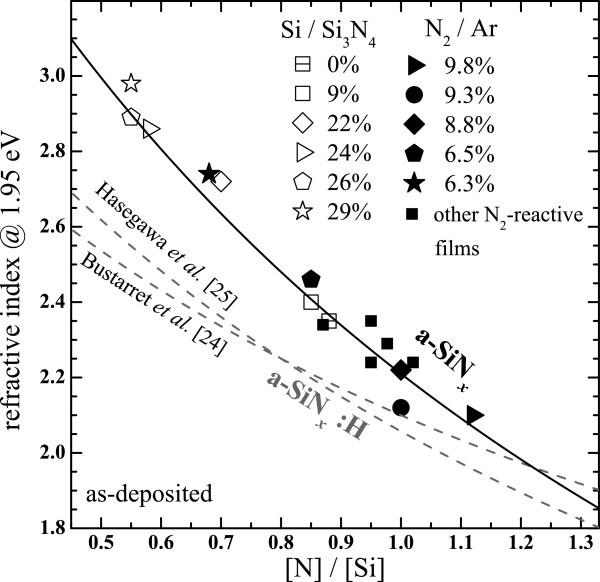
**Evolution of the refractive index of SiN**_***x***_**thin films.** The films were produced by the N_2_-reactive and the co-sputtering methods as a function of [N]/[Si] ratio. The data are compared with a new model (black curve) and with two models (dashed curves) but concerning hydrogenated films.

Figure 3 shows the evolution of the refractive index of SiN_*x*_ films (given at 1.95 eV) produced by the two methods as a function of the [N]/[Si] ratio *x*. The numerous results show that *x* progressively increases independently of the synthesis method with increasing either Ar/N_2_ or Si/Si_3_N_4_. Bustarret et al. [24] proposed that the refractive index *n* could be represented as the bonding-density-weighted linear combination of reference refractive indexes taken at *x* = 0 and *x* = 4/3 using the empirical relation:

(1) $$x=4/3\left(n_{a-\mathrm{si}}-n\right)/\left(n+n_{a-\mathrm{Si}}-2n_{a-Si_{3}N_{4}}\right)$$

Nevertheless, one can notice in Figure 3 that our experimental results progressively diverge from the models obtained by this group and also by Hasegawa et al. [25] while *x* is decreased. However, the two groups both studied hydrogenated SiN_*x*_ films (SiN_*x*_:H) in contrast to our results. Besides, these latter authors have shown that the Si-H density increased while *x* was experimentally decreased. Consequently, the drop of *n* is explained by the H incorporation in their material as suggested elsewhere [26]. However, we could use this model to fit the experimental data but using the refractive index of a-Si (*n*_a-Si_ = 4.37, see Figure 2) instead of hydrogenated a-Si (*n*_a-Si:H_ = 3.3) used by Bustarret et al. [24]. This shows again the influence of H on the optical properties of the films. We obtained $n_{a-{Si}_{3}N_{4}}$ = 1.85, which is similar to many previous results [25-27], but is lower than 2.03 that is commonly used for a-Si_3_N_4_[28]. This difference could be explained by the incorporation of voids in the microstructure [27] as attested by the presence of residual Ar atoms detected by RBS in the as-deposited films. Besides, this explanation is confirmed by the density *ρ*_*v*_ of our SiN_*x*_ films which was calculated using the atomic areal density *ρ*_*s*_, and the film thickness *d*, obtained by RBS and ellipsometry analyses, respectively, with the following relation: *ρ*_*v*_ = *ρ*_*s*_ / *d*. We found that the density varied from 2.4 to 2.8 g/cm^3^, which is again sensibly lower than that of a-Si_3_N_4_ of 3.1 g/cm^3^ reported in the literature [29].

Considering the RBS and the ellipsometry spectra, we have produced thin SiN_*x*_ films with various compositions that do not depend on the synthesis method, but only on the Si content. As a consequence, *n* is a precise indicator of the composition that will be used in the following sections.

### FTIR

**Figure 4:**
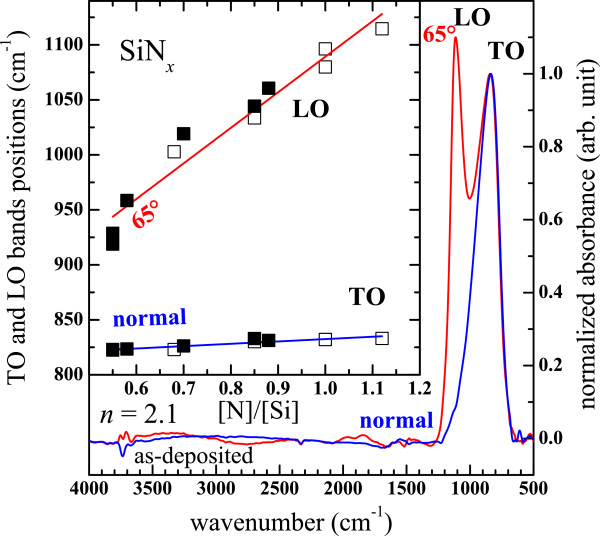
**FTIR spectra of a SiN**_***x***_**thin film.** The films were deposited by the N_2_-reactive method recorded with a normal incidence and with an incidence angle of 65°. The inset shows the TO and LO band positions of SiN_*x*_ layers deposited by the N_2_-reactive (full squares) and the co-sputtering (empty squares) methods as a function of the composition.

Figure 4 shows the typical FTIR spectra of a SiN_*x*_ film with a low refractive index of 2.1 (SiN_1.12_) which were recorded with a normal incidence and with an incidence angle of 65°. One can observe only one absorption band centered at 833 cm^−1^ in the spectrum measured with the normal incidence, whereas an additional shoulder at 1115 cm^−1^ emerged while the incidence angle was changed to 65°. Moreover, it is essential to note that no other absorption bands were discernible in the 700 to 4000 cm^−1^ spectral range whatever the deposition approach. No Si-O absorption bands (transverse optical (TO_4_) at 1200 cm^−1^, longitudinal optical (LO_4_) at 1160 cm^−1^, TO_3_ at 1020 to 1,090 cm^−1^, LO_3_ at 1215 to 1260 cm^−1^, TO_2_ at 810 cm^−1^, and LO_2_ at 820 cm^−1^) [30,31] were detected in all spectra. This demonstrates the absence of O contamination that has been also confirmed by RBS and the absence of any oxide layers at the interface between the Si-wafer and the SiN_*x*_ layers which has been confirmed by HRTEM observations. Besides, no absorption bands of Si-H stretching mode in the 2090 to 2200 cm^−1^ spectral domain were detected because of our synthesis methods involving no hydrogen. Since the latter band is generally the most intense Si-H vibration mode observed in SiN_*x*_:H, one can then conclude on the absence of the Si-H wagging (630 to 650 cm^−1^) and asymmetric stretching (840 to 900 cm^−1^) modes in the spectra [24,25,27,32-34]. In the same manner, no absorption bands of N-H stretching mode were detected in the 3320 to 2500 cm^−1^ spectral region suggesting that the N-H bending (1140 to 1200 cm^−1^) modes are also absent in our spectra [24,25,32,33]. As a consequence, the 833-cm^−1^ band and the 1115-cm^−1^ shoulder can be unambiguously assigned to the transverse (TO) and the longitudinal (LO) modes of the asymmetric Si-N stretching vibration, respectively [24,33-37]. The TO-LO splitting is due to the Berreman effect [38] according to which only the TO mode is IR active in normal incidence, and the shoulder observed with an incidence angle of 65° corresponds to the LO mode. Then, the analysis of the FTIR spectra in the 700 to 1200 spectral domain is particularly interesting since it definitely concerns the Si-N bonding alone, in contrast to many works on the FTIR study of SiN_*x*_:H films [5,27,32-34,39], Si nitride layers containing oxygen [19,20], or SiN_*x*_ layers stacked between Si oxide layers [17,40].

**Figure 5:**
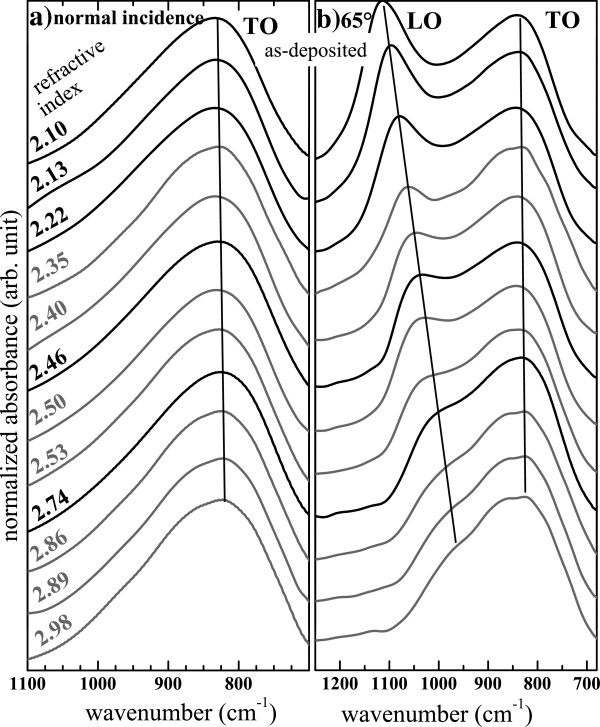
**Evolution of the FTIR spectra of SiN**_***x***_**with the refractive index.** The FTIR spectra of the layers deposited by the N_2_-reactive (black) and the co-sputtering (gray) methods were measured with a normal incidence (**a**) and with an incidence angle of 65° (**b**).

Figure 5 shows the evolution of the FTIR spectra of SiN_*x*_ thin films measured with the two incidence angles. The spectra are arranged with increasing order of *n* of SiN_*x*_ films deposited by both methods. One can notice that the evolution of the FTIR spectra is not influenced by the deposition method but only by the composition. The spectra in Figure 5a showing the TO band only change slightly with *n*, whereas the evolution of the spectra in Figure 5b is more pronounced because of the significant blueshift of the LO band and the concomitant increase of its intensity with decreasing *n*. The TO band shifts to higher wavenumbers as well but with a lesser extent.

Similar blueshifts of the TO band [5,25,27,32-34] and of the LO band [24,27,33] were also observed in SiN_*x*_:H films. Lucovsky et al. [32] explained the TO band blueshift by the incorporation of H. They suggested that one of the near silicon atoms of the N(−Si)_3_ bonding configuration where Si has only one N neighbor is replaced by a H atom. The H incorporation was also evoked to be responsible for the LO band blueshift in SiN_*x*_:H [24,27,33,39]. However, our spectra in Figure 5 demonstrate that these two blueshifts are not necessarily linked to H. Besides, similar blueshifts of the TO band [15,35] and of the LO band [35] have also been reported in O- and H-free SiN_*x*_ thin films while the Si content was decreased. As a consequence, these two blueshifts are partly or completely due to some change of the [N]/[Si] ratio in the case of SiN_*x*_:H or pure SiN_*x*_, respectively. The change in the positions of the TO and the LO modes of Si-N absorption bands are due to some modifications intrinsic to the Si-N binding configuration. In their calculation, Hasegawa et al. [25] have predicted that the blueshift of the TO mode is linked to the decrease of the Si-N bond length which is caused by a compositional change of SiN_*x*_[25,41]. In addition to this, some stress in the films induced by the Si incorporation may also contribute to such shifts [35]. Moreover, one can assume that the TO-LO coupling of the Si-N asymmetric stretching modes is induced by the disorder in the material in the same manner as that established in Si oxide [42,43]. Consequently, the increase of the LO band intensity is a signature of the ordering of the films while the Si content is decreased.

The inset of Figure 4 shows the TO and LO band positions as a function of the stoichiometry. Again, one can notice that the LO band position is more sensitive to the composition than that of the TO band. The LO mode position is obviously a better indicator of the composition of Si-rich SiN_*x*_ than that of the TO band, as mentioned elsewhere [35]. We found that the TO and the LO band positions increase linearly with increasing Si/N ratio *x* following the two relations:

(2) $$\nu_{\mathrm{TO}}\left(x\right)=21.4\left(x-4/3\right)+\nu_{\mathrm{TO}}\left(4/3\right)$$

(3) $$\nu_{\mathrm{LO}}\left(x\right)=323.4\left(x-4/3\right)+\nu_{\mathrm{LO}}\left(4/3\right)$$

where *ν*_TO_(*x*) and *ν*_LO_(*x*) are the TO and the LO band positions, respectively, and *ν*_TO_(4/3) and *ν*_LO_(4/3) are the TO and the LO band positions calculated for *x* = 4/3, which correspond to the stoichiometric condition, respectively. We found *ν*_TO_(4/3) = 840 cm^−1^ which is interestingly the value attributed to the Si-N stretching vibration of an isolated nitrogen in a N-Si_3_ network [33,44] and *ν*_LO_(4/3) = 1197 cm^−1^. These relations can be used to estimate the composition of as-deposited Si-rich SiN_*x*_ films in the same way as the empirical one concerning Si-rich silicon oxide [30].

**Figure 6:**
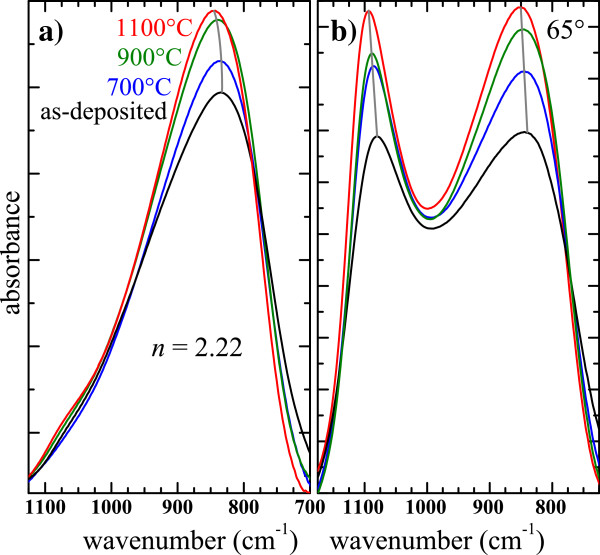
**Effect of the annealing temperature on the FTIR spectra of SiN**_***x***_**.** The FTIR spectra were recorded under normal incidence (**a**) and with an angle of 65° (**b**).

In Figure 6a, the effect of the annealing on the FTIR spectra of a SiN_*x*_ film with *n* = 2.22 is shown. It is seen that the intensity of the TO mode increases with increasing annealing temperature which is manifestly due to the increase in the amount of Si-N bonds. It is also seen that the TO peak position slightly shifts to higher wavenumbers. Moreover, Figure 6b shows that the LO band evolves similarly, *i.e.*, an increase of its intensity and a significant blueshift. All these observations indicate a rearrangement of the Si nitride network toward that of the stoichiometric structure with a lower structural disorder. This can be due to a phase separation between Si and Si nitride.

### Raman spectroscopy

**Figure 7:**
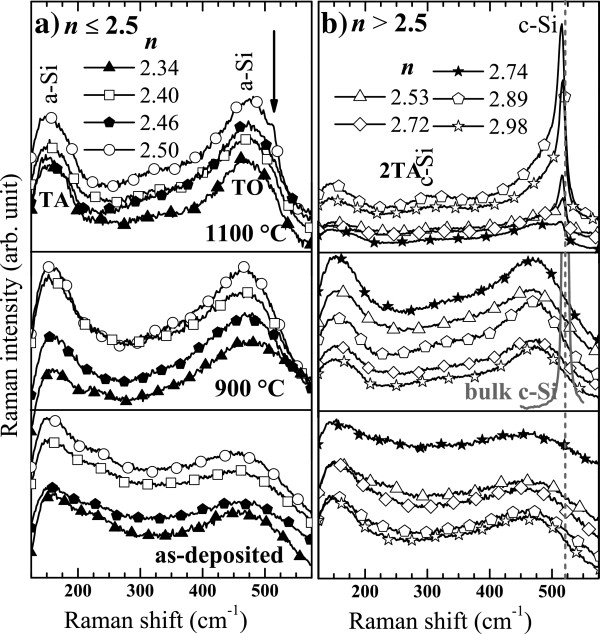
**Evolution of the Raman spectra of SiN**_***x***_**with the refractive index and the annealing temperature.** Effect of the annealing temperature on the Raman spectra of SiN_*x*_ thin layers deposited on fused silica with a refractive index below 2.5 (**a**) and above (**b**). It independently concerns films produced by the N_2_-reactive (full symbols) and the co-sputtering (empty symbols) methods. The excitation power density was 0.46 MW/cm^2^.

Figure 7 shows the evolution of the Raman spectra of SiN_*x*_ thin layers deposited on fused silica with various Si contents and with various annealing temperatures. Again, it is seen that the evolution of the Raman spectra does not depend on the deposition methods but only on the composition that is set by *n*. Upon annealing at 900°C, the two broad vibration bands of the transverse acoustic (TA) phonon and of the TO phonon of a-Si at 150 and 480 cm^−1^, respectively, became clearly narrower and more pronounced (Figure 7). This evolution can be explained by the formation of small amorphous Si-np [45]. Unlike this deduction, the appearance of new sharp peaks slightly shifted towards lower wavenumbers compared to bulk crystalline Si (c-Si) at approximately 520 cm^−1^ upon annealing at 1100°C as shown in Figure 7b, which unequivocally demonstrates the formation of small crystalline Si-np. Besides, the formation of a c-Si phase is also consistent with the appearance of a weak peak at 300 cm^−1^ that is attributed to the second order of the transverse acoustic (2TA) phonon mode in the thin films containing a high Si content (*n* = 2.89 and 2.98). It is seen that the condensation of the excess of Si in small crystalline Si-np during the annealing at 1100°C occurs but only in thin films having a refractive index higher than 2.5 (Figure 7b) or maybe equal to 2.5 as indicated by the presence of a weak shoulder (see the arrow) in Figure 7a. Nevertheless, thin films with a low Si content (SiN_*x* > 0.8_, see Figure 3) could also contain small Si-np upon annealing at 1100°C but having an amorphous structure.

**Figure 8:**
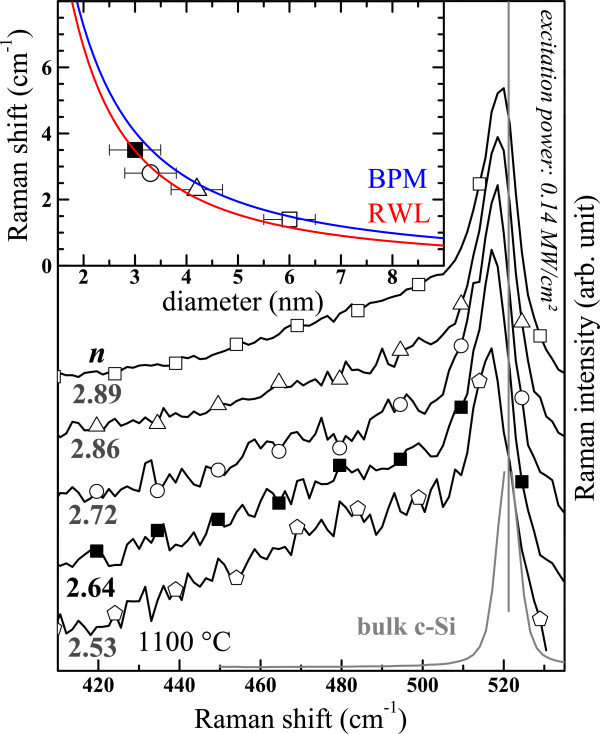
**Crystalline Si peaks in Raman spectra of SiN**_***x***_**films for various refractive indexes.** Raman spectra of the films produced by the N_2_-reactive and the co-sputtering methods are displayed with empty and full symbols, respectively. The inset shows the Raman frequency redshift as a function of the crystalline Si-np average size measured by HRTEM. The curves of the RWL and BP models are shown for comparison.

**Figure 9:**
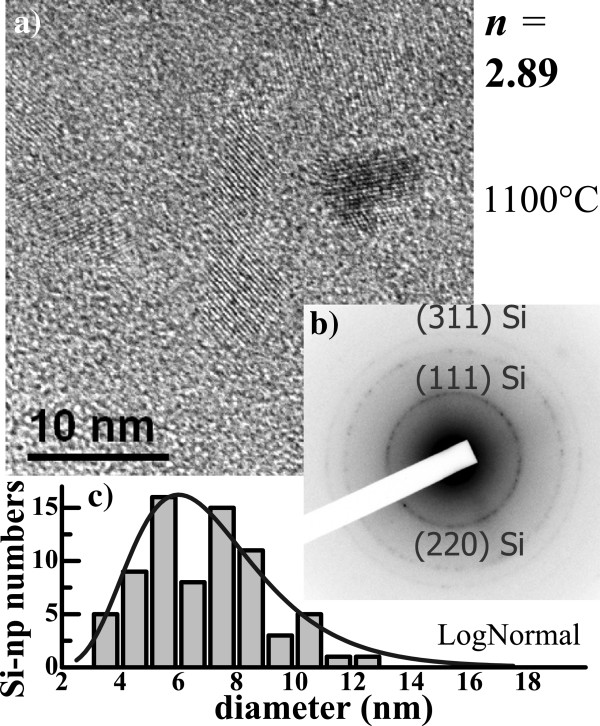
HRTEM image (a), diffraction pattern (b), and Si nanocrystal size distribution (c).

Figure 8 shows the Raman spectra of the thin films with *n* > 2.5 (Figure 7b) after annealing at 1100°C. A low excitation energy density of 0.14 MW/cm^2^ was used to record these spectra in order to avoid any heating and induced stress of the films that may affect the Raman spectra of crystalline Si-np [46]. One can observe that the c-Si peaks progressively shift to higher wavenumbers toward the peak position of bulk c-Si with increasing *n*. This progressive shift is related to a QCE on the optical phonon in confined crystalline Si-np [46-49], as it is seen in the inset of Figure 8 where the Raman shift is plotted as a function of the diameter. The Raman shift was obtained by fitting the Raman signal with the asymmetric Lorentzian functions, and the particle size corresponded to the maximum of the lognormal distribution of crystalline Si-np sizes measured by HRTEM (see Figure 9). Then, we compared our experimental results with the Richter, Wang, and Ley (RWL) model [47] and the bond polarizability (BP) model [48] that account for the QCE on optical phonons in crystalline Si-np. In these two models, the Raman redshift can be presented as a function of the Si-np size using the analytical expression:

(4) $$\Delta \omega =\beta {\left(a/d\right)}^{\gamma }$$

where Δ*ω* is the frequency redshift; *a*, the Si lattice parameter (*a* = 0.543 nm); *d*, the crystalline Si-np diameter; and *β* and *γ*, the model parameters (*β* = 52.3 cm^−1^ and *γ* = 1.586 for the RWL model, and *β* = 47.41 cm^−1^ and *γ* = 1.44 for the BP model). Interestingly, one can notice that our experimental results are in good agreement with the previous works suggesting that the latter models can be applied to crystalline Si-np embedded in Si nitride as well.

### HRTEM

In order to further investigate the microstructure of the 1100°C-annealed films, HRTEM observations have been performed on several thin films with various *n* > 2.5. Figure 9b shows the diffraction pattern of one film with *n* = 2.89. One can observe three quasi-continuous rings corresponding to various orientations of c-Si because of the presence of randomly oriented crystalline Si-np. These numerous crystalline Si-np can be easily distinguished from the host matrix (Figure 9a) because of the lattice fringes of c-Si. They are rather small with an average size of about 6.0 ± 0.5 nm (Figure 9c).

### XRD

**Figure 10:**
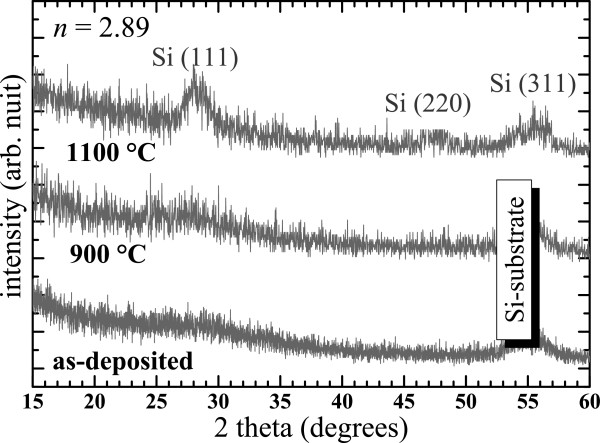
**Evolution of the XRD pattern of a SiN**_***x***_**layer as a function of the annealing temperature.**

Figure 10 shows the effect of the annealing temperature on the XRD patterns of one SiN_*x*_ layer produced by the co-sputtering method with *n* = 2.89. One can observe that two new peaks of c-Si with the (111) and (220) orientations distinctly emerge in the XRD pattern upon annealing at 1100°C, which demonstrates the formation of a c-Si phase in the material.

**Figure 11:**
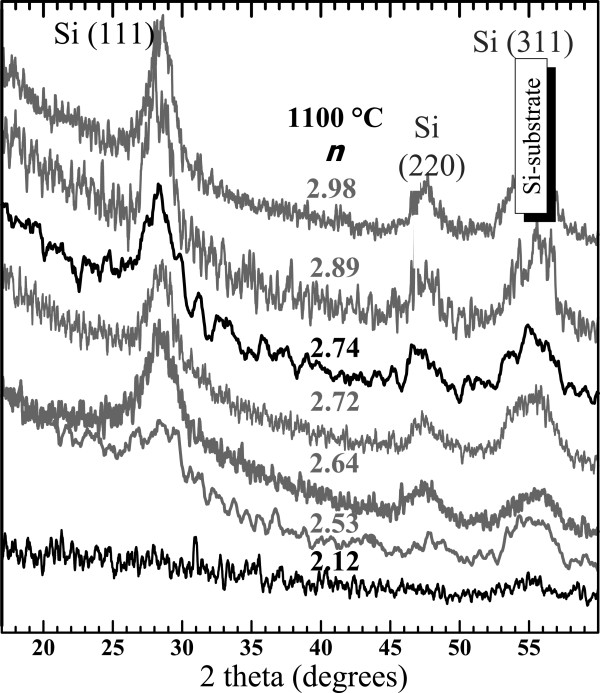
**Evolution of XRD pattern of 1100°C-annealed SiN**_***x***_**layers with the refractive index.** XRD curves of thin films produced by the N_2_-reactive and the co-sputtering methods are displayed in black and gray, respectively.

In Figure 11, the evolution of the XRD pattern of the 1100°C-annealed films with *n* is shown. Again, we could not notice any dependence of the synthesis method on the structure since the spectra of the two films produced by the co-sputtering and the N_2_-reactive methods which have a close refractive index of 2.72 and 2.74, respectively, are very similar. The XRD patterns depend only on the Si content given by *n*. One can notice that the thin films with *n* = 2.12 do not show any c-Si peak with the exception of the (311) c-Si peak emanating from the substrate. This is in contrast with the spectra of thin films with a higher refractive index (*n* > 2.5) that also show the (111) and (220) c-Si diffraction peaks attesting the presence of crystalline Si-np. Besides, the XRD results are in perfect agreement with the Raman spectra shown in Figure 7, since the c-Si Raman peaks were also detected but only when *n* was above 2.5 (SiN_*x*<0.8_).

### Photoluminescence

**Figure 12:**
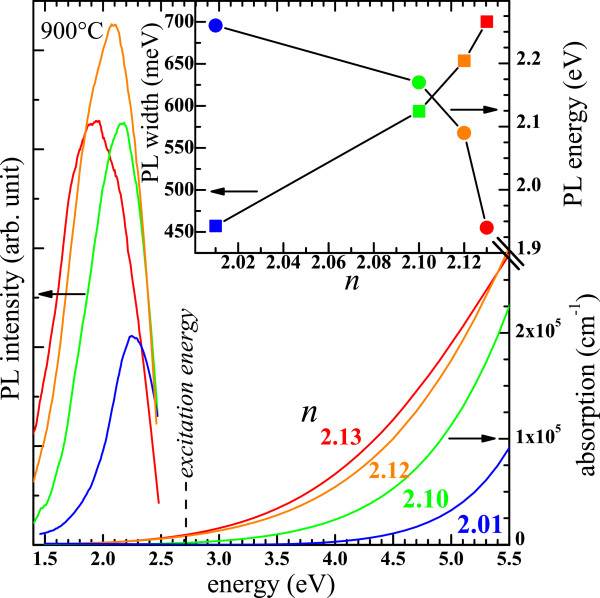
**Variations of the PL and the absorption spectra with the refractive index *n*.** The inset shows the evolution of the peak position and the band width with *n*.

**Figure 13:**
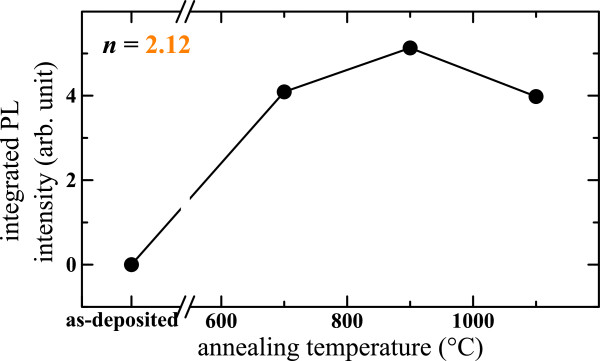
Evolution of the integrated PL intensity with the annealing temperature.

Figure 12 shows the PL and the absorption spectra of several SiN_*x*_ thin films with various *n*. In the right part of the figure, it is seen that the absorption rises with increasing *n* which is explained by the increase of the Si content. In the same time, we observed a progressive redshift of the PL bands with a concomitant increase of their widths as displayed in the inset. Moreover, one can notice that the PL intensity significantly increases while *n* increases from 2.01 to 2.12, which is partly explained by the rise of the absorption. Reminding that FTIR spectra showed that the disorder increased with increasing *n*, the increase of the non-radiative recombination rate would then explain the decrease of the PL intensity while *n* reaches 2.14. Besides, thin films with *n* > 2.4 (SiN_*x*<0.85_) did not exhibit any PL even after annealing with various temperatures ranging up to 1100°C. The typical variation of the PL intensity of one luminescent film with the annealing temperature is shown in Figure 13. Interestingly, as-deposited films showed no PL, and it is seen that the highest integrated PL intensity was found at 900°C. The origin of the visible PL easily perceivable by the naked eye is investigated in the ‘Discussion’.

### Laser annealing

**Figure 14:**
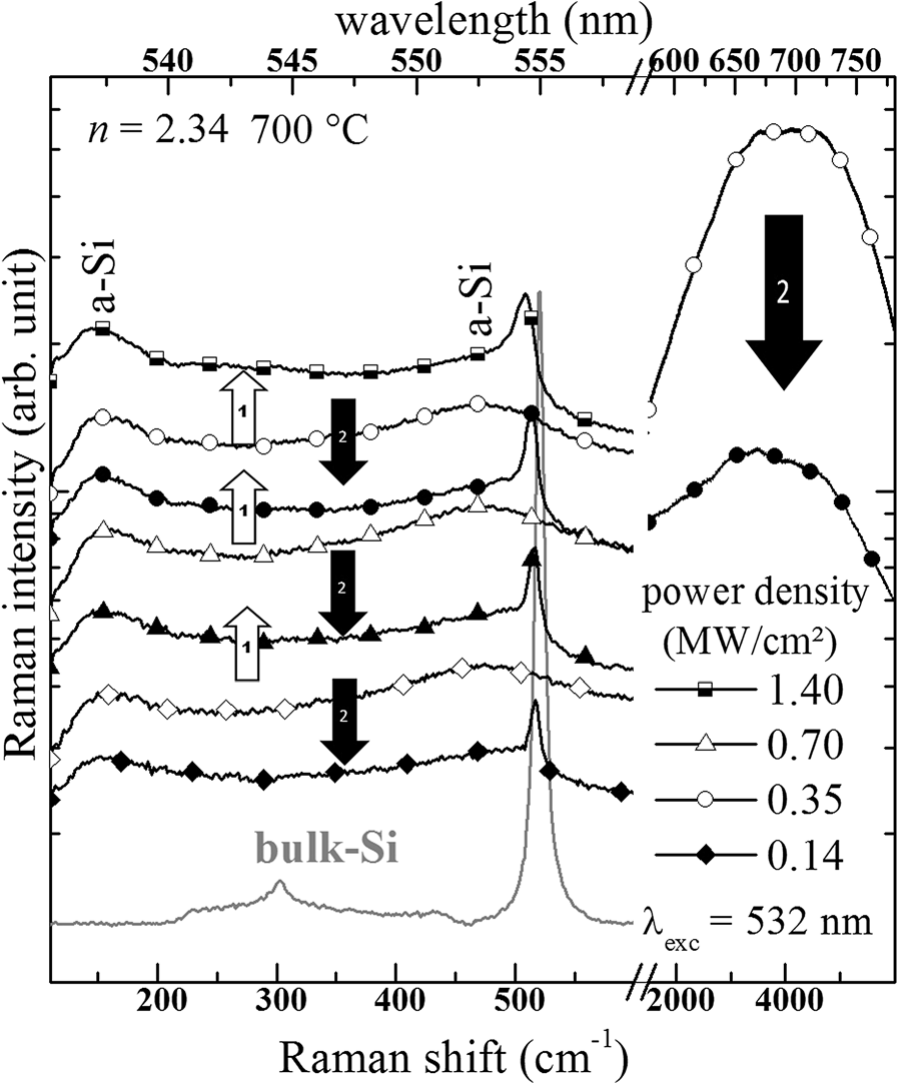
**Laser annealing effect on the Raman spectra of SiN**_***x***_**films deposited on fused silica substrates.**

Figure 14 shows the Raman spectra of one luminescent film with *n* = 2.34 recorded with various excitation power densities. Although we did not detect by Raman spectroscopy (Figure 7a) any crystalline Si-np even after annealing at 1100°C, we could however form small Si nanocrystals by laser annealing. This formation has been evidenced by Raman measurements that are separated in two steps for clarity. During the first step (white arrows), the power density of the laser was increased from 0.14 to 0.70 MW/cm^2^. It is seen that the Raman signal increased because of the rise of the number of both Raman-scattered photons and PL photons. When the excitation power density reached 1.4 MW/cm^2^, a sharp new Raman peak slightly shifted from that of bulk c-Si appeared. During the second step (black arrows), the power density was decreased back. One can observe that the c-Si peak remained whatever the power density suggesting that the structure of the SiN_*x*_ thin layer was definitively modified. This is then explained by the formation of small crystalline Si-np in the spot of the focused laser as observed elsewhere [45,50,51]. Moreover, one can notice that, for the same excitation densities, all baselines levels significantly dropped after the local formation of small Si nanocrystals. This drop of the baseline level is explained by the PL quenching of the broad PL band centered at about 700 nm, corresponding to approximately 4000 cm^−1^, since the baseline is actually located on the green tail of the broad PL band. This demonstrates that this PL band cannot emanate from crystalline Si-np. This PL could however be related to amorphous Si-np. Nevertheless, Volodin et al. [45] showed that the presence of amorphous Si-np is not required for the laser-induced formation of crystalline Si-np which is in agreement with our results showing that this formation occurred in films containing a low Si content (SiN_0.9_) and in as-deposited films as well.

**Figure 15:**
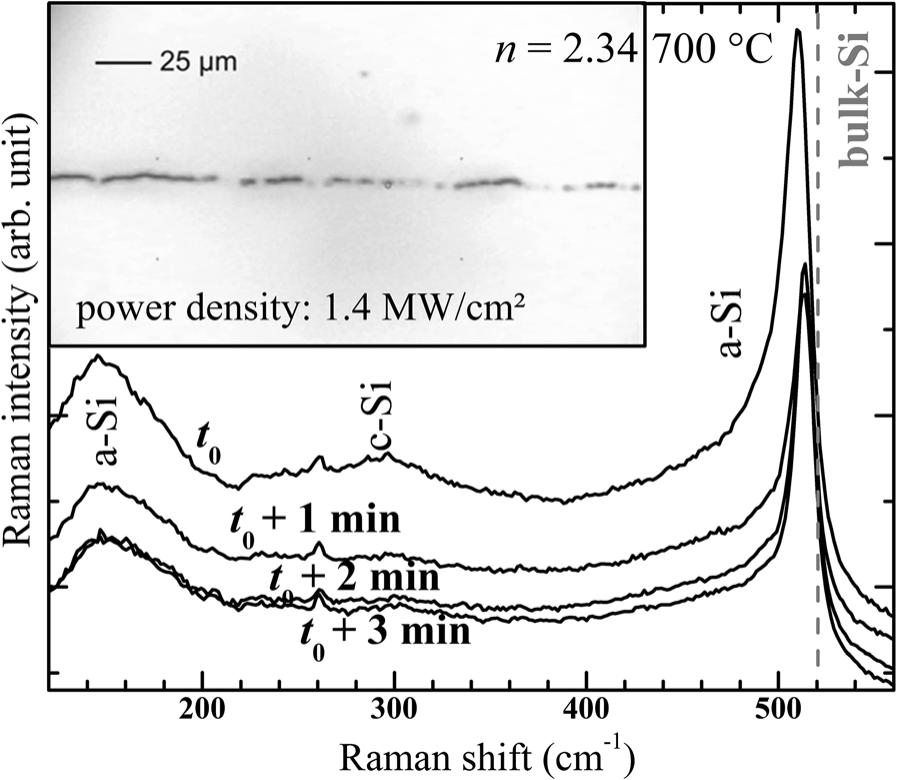
**Effect of the irradiation duration on the Raman spectra of SiN**_***x***_**films during the laser annealing.** The inset shows the picture of the laser spot course on the SiN_*x*_ layer.

Figure 15 shows the effect of the irradiation time on the Raman spectra of the latter SiN_*x*_ films during the laser annealing which was performed while the power density was set to 1.4 MW/cm^2^ (Figure 14). The formation of small crystalline Si-np is very fast since the c-Si peaks at 300 and 510 cm^−1^ emerged almost immediately or at least in less than the acquisition time of approximately 0.5 s after the laser irradiation started. Moreover, one can observe that, after the laser-induced formation of crystalline Si-np, the Raman spectra changed while the thin SiN_*x*_ layer was continuously exposed to the intense radiation. Indeed, three modifications are clearly seen: (1) The baseline progressively dropped with increasing irradiation time which has been previously explained by the PL quenching of the material (see Figure 14). (2) The c-Si peak of 7.5 cm^−1^ shifted towards the position of c-Si in bulk material, and its intensity dropped after 1 min. However, its position and its intensity remained fixed for longer irradiation times. This latter modification, which is actually also discernible in Figure 14, can be explained by the unceasing growth of the crystalline Si-np until they reached a maximal size and/or by the relaxation of stress [46]. Also, (3) the intensity of the 2TA phonon mode at 300 cm^−1^ was quenched after 1 min of laser exposure which may result from disorder in the crystalline structure [52]. Besides, in the inset of Figure 15, a picture of the laser spot course while the thin layer was displaced perpendicularly to the laser beam is shown. This dark laser print reveals some local damages caused by the long exposition. However, since the main peak remains shifted to lower wavenumbers compared with bulk c-Si after a long illumination, one can assure that the film structure was definitively modified and that the films contained crystalline Si-np locally formed by laser annealing.

## Discussion

The extensive investigation of the microstructure of SiN_*x*_ films versus the composition and the annealing treatments enables us to discuss on the PL origin considering that the films do not contain any oxygen and hydrogen. We show that neither defect states within the bandgap nor band tail states could account for all the aspects of the PL. Although we could form crystalline Si-np, we show that the radiative emission is not originating from confined states in crystalline Si-np but could be related to small amorphous Si-np.

### Defect states in the bandgap

Optically active defect states within the bandgap of amorphous SiN_*x*_ could play a role in the radiative recombination of SiN_*x*_ as reported by several authors [18,53]. This interpretation is based on the wide PL spectra that contained distinct PL peaks with several energy levels that corresponded to the calculated values of various defect states found by Robertson [54,55]. Similar spectra were observed in the 1.75 to 3.1 eV spectral range by Ko et al. [56] who noticed a redshift of the PL with decreasing Si content. This evolution is in contrast to that of our PL spectra which, moreover, do not contain any distinct PL peaks attributable to distinct defect state levels. As a consequence, we believe that the origin of the PL of our SiN_*x*_ samples cannot be ascribed to defect states localized within the bandgap.

### Band tail recombination (static disorder model)

Let us consider the optical transition between photogenerated carriers localized in the band tail of the material in accordance with the static disorder model [57]. In this model, the carrier distribution in the exponential band tail density of states accounts for the PL band position and the PL shape of SiN_*x*_:H [16]. An increase of the width of the localized states results in a blueshift and an increase of the width of the PL band. On one hand, many groups [13,16] explained that the increase of the structural disorder caused by the nitrogen alloying in Si-rich SiN_*x*_:H with a very high Si content (SiN_*x*<0.6_) accounts for the widening of the band tail states and then for the PL behavior. On the other hand, many groups [2-4] explained that the increase of the structural disorder induced by the incorporation of more nitrogen in N-rich SiN_*x*>1.33_:H films accounts for the widening of the band tails and the PL properties. The increase of disorder in N-rich SiN_*x*>1.33_ films with increasing N content is consistent with the FTIR spectra of Huang et al. [35] which show a drop of the LO band intensity.

Figure 5b showed that the situation is inversed in our Si-rich SiN_*x*_ films with a low Si excess content since the disorder manifestly increases with the Si incorporation. Therefore, the redshift of the PL band (Figure 12) cannot be explained by the tail-to-tail radiative recombination which would anyway be in contradiction with the widening of the PL band (inset of Figure 12). As a consequence, unlike Si-rich SiN_*x*_:H films with a very high Si content (SiN_*x*<0.6_) [13,16], we believe that the static disorder model cannot account for the PL properties of H-free Si-rich SiN_*x*_ films containing a low Si content (SiN_*x*>0.85_). Besides, it has been shown that the hydrogen concentration plays an important role in the PL properties (intensity and peak position) of hydrogenated films [13].

### Crystalline Si-np

Crystalline Si-np were detected by Raman, XRD, and HRTEM in numerous SiN_*x*_ films annealed at 1100°C that had a high *n* > 2.5 (SiN_*x*<0.8_). Furthermore, we have demonstrated in Figure 8 that the progressive redshift of the crystalline Raman peak while *n* decreased is due to the decrease of the crystalline Si-np average size. The average sizes in the films with *n* ranging from 2.53 to 2.89 are between 2.5 and 6 nm, respectively. Theses sizes are theoretically small enough to show PL from excitons confined in crystalline Si-np according to the QCE model [58]. This model was proposed to explain the size dependence of the PL peak position that was noticed in oxide systems [1,59]. This size effect was evidenced in free crystalline Si-np surrounded by a thin Si oxide shell [60], which however slightly differ from that generally observed while the crystalline Si-np are embedded in a Si oxide host medium [59,61]. In the case of Si nitride as embedding matrix, several authors suggested that the PL could emanate from confined states in crystalline Si-np, which were present in the materials as attested by HRTEM observations, mainly because of a perceivable size effect on the PL [10-14]. Although our measurements (Figure 12) also show that the PL peak shifted to lower energies with increasing Si content, which is consistent with the QCE model, crystalline Si-np cannot be responsible for the radiative emission for two reasons: (1) Although small (2.5 to 6 nm) Si nanocrystals could be formed in films with *n* > 2.5 during annealing at 1100°C, we could not detect any PL. PL was detected only for smaller refractive indexes (*n* < 2.4). Besides, we demonstrated in Figure 7b and Figure 10 that this temperature is necessary to crystallize the excess of Si. Furthermore, (2) the PL of luminescent SiN_*x*_ films (*i.e.*, with *n* < 2.4) was quenched while we could form crystalline Si-np by another annealing method using an intense laser irradiation (Figure 14).

### Amorphous Si-np

Although we have demonstrated that crystalline Si-np are not valid to explain the PL, let us consider Si-np with an amorphous phase as proposed by several authors [5-8]. The annealing temperature dependence of the FTIR spectra of one luminescent SiN_*x*_ film (*n* = 2.22) shown in Figure 6 suggests that a phase separation between Si-np and the Si nitride host media occurred during the annealing. The two Raman bands of a-Si at 150 and 485 cm^−1^ shown in Figure 7 indicate that luminescent films (*i.e.*, with *n* < 2.4) could contain amorphous Si-np. Besides, the Raman spectra would then show that the density of amorphous Si-np increased with increasing annealing temperature. This explains the absence of PL in the as-deposited samples and why the highest integrated PL intensity (Figure 13) was found at 900°C and not at 1100°C when crystalline Si-np could form. The redshift of the PL bands with increasing Si content (Figure 12) would then be due to a size effect. Also, the increase of the PL band width would then result from the widening of the size distribution as experimentally observed in Si oxide matrices [59,61]. Then, we have imaged a 1,000°C-annealed SiO_*x*_/SiN_*x*_ multilayer by energy-filtered transmission electron microscopy enabling to distinguish small amorphous Si-np from the host media because of the high contrast of this technique. Because of PL interest, the refractive index of the SiN_*x*_ sublayer was set between 2.1 and 2.3. We could distinctly observe amorphous Si-np in the 3.5-nm-thick SiO_*x*_ sublayers, but no particles were perceivable in the 5-nm-thick SiN_*x*_ sublayers [40]. Si-np could be however very small, below the EFTEM detection threshold of about 1 to 2 nm, and then constituted less than 1000 of Si atoms. Besides, such an amorphous Si-np size seems possible compared to the average size of 2.5 nm of crystalline Si-np detected by Raman spectroscopy in SiN_*x*_ with *n* = 2.53. Consequently, the origin of the PL would be related to small amorphous Si-np, and the recombination would originate either from confined states in the Si-np and/or from defect states at the interface between the Si-np and the Si nitride medium [7].

## Conclusion

We have produced pure amorphous Si-rich SiN_*x* < 1.33_ thin films by magnetron sputtering with various Si contents using two deposition methods, namely the N_2_-reactive sputtering of a Si target and the co-sputtering of Si and Si_3_N_4_ targets. The dependence of the only Si content on the microstructure and on the optical properties was studied. The two synthesis methods are equivalent since no systematic change could be discerned in the structural and the optical analyses. Besides, no trace of O atoms was detected by RBS and by FTIR, and no H bonded to Si or N could be detected by FTIR. We could then establish an empirical relation between the [N]/[Si] ratio and *n* based on the random bonding model on pure SiN_*x*_ which manifestly differs from previous relations that concerned SiN_*x*_:H because of the H incorporation induced by the chemical deposition techniques.

Because of the absence of Si-H, N-H, and Si-O absorption bands, we could highlight the Berreman effect on the FTIR spectra of SiN_*x*_ by the normal incidence and an oblique illumination. The TO-LO pair modes of the two Si-N stretching absorption bands could be unambiguously assigned. A redshift of the two modes and a drop of the LO band intensity were observed while the Si content increased, which indicates that incorporation of more Si generates more disorder in the films. Moreover, a significant blueshift of the two modes with increasing annealing temperature was noticed which may be explained by a phase separation between Si-np and the Si nitride medium. At the same time, the LO band intensity increased indicating a rearrangement of the Si nitride network towards less disorder.

The effect of the annealing temperature on the Raman spectra has been investigated on films with *n* < 2.5 (SiN_*x*>0.9_). The Raman spectra indicate that small amorphous Si-np could be formed during the annealing and that their density increased with the annealing temperature. For higher *n* (*n* > 2.5, SiN_*x*<0.8_), Raman spectra, as well as XRD patterns, demonstrated that crystalline Si-np are formed upon annealing at 1100°C. Moreover, QCE on the optical phonon in crystalline Si-np embedded in Si nitride was observed. It matches with previous theoretical models concerning Si nanocrystals in Si oxide systems. The average size measured by HRTEM increased from 2.5 to 6 nm with increasing *n*.

Only SiN_*x*_ films with *n* ranging from 2.01 to 2.34 (SiN_*x*>0.9_) exhibit visible PL. The PL bands redshifted and widened while *n* was increased. The tail to tail recombination cannot account for these PL properties since the FTIR spectra showed that the disorder increased with increasing *n* which would result in a blueshift and a widening of the PL bands. The PL could be then due to a QCE. The annealing temperature dependence of the PL intensity is consistent with the formation of Si-np. Nevertheless, the PL is not related to crystalline Si-np since they have not been detected in luminescent films by XRD and Raman measurements. As an additional proof, the PL quenched while Si crystalline Si-np could be formed by an intense laser irradiation. As a consequence, we believe that the PL is actually related to small amorphous Si-np and/or defect states that could be located at the interface between Si-np and the Si nitride host medium.

## Competing interests

The authors declare that they have no competing interests.

## Authors’ contributions

OD wrote the article and carried the interpretation of the data. OD produced the samples and characterized them by spectroscopic ellipsometry, FTIR, absorption, PL, and Raman. JP carried out the RBS measurements. XP investigated the structure by HRTEM. SPN produced the multilayers. JC has been involved in the discussion about the origin of the PL. FG proposed and guided the project. All authors read and approved the final manuscript.
